# DDX5-targeting fully human monoclonal autoantibody inhibits proliferation and promotes differentiation of acute promyelocytic leukemia cells by increasing ROS production

**DOI:** 10.1038/s41419-020-02759-5

**Published:** 2020-07-20

**Authors:** Jing Wu, Yan-Qiu You, Yan-Xiu Ma, Yan-Hua Kang, Tian Wu, Xiang-Ji Wu, Xiao-Xiao Hu, Qiao-Hong Meng, Yin Huang, Na Zhang, Xiao-Ben Pan

**Affiliations:** 1 Department of Basic Medicine of Medical School, Department of Infectious Diseases of Affiliated Hospital, Institute of Liver and Metabolic Diseases, Key Laboratory of Aging and Cancer Biology of Zhejiang Province, and Key Laboratory of Inflammation and Immunoregulation of Hangzhou, Hangzhou Normal University, 310000 Hangzhou, Zhejiang P.R. China; 2 Department of Laboratory Medicine, The Second Affiliated Hospital of Harbin Medical University, 150001 Harbin, Heilongjiang P.R. China; 3 Peking University People’s Hospital, Peking University Hepatology Institute, Beijing, P.R. China

**Keywords:** Cancer therapy, Diseases

## Abstract

Acute promyelocytic leukemia (APL) therapy involves the compounds cytotoxic to both malignant tumor and normal cells. Relapsed APL is resistant to subsequent chemotherapy. Novel agents are in need to kill APL cells selectively with minimal toxicity. DDX5 has been recognized to be a novel target to suppress acute myeloid leukemia (AML). However, the role of DDX5 remains elusive in APL. Here a DDX5-targeting fully human monoclonal autoantibody named after 2F5 was prepared. It is demonstrated that 2F5 selectively inhibited APL cell proliferation without toxicity to normal neutrophil and tissues. Moreover, 2F5 was confirmed to induce G0/G1 phase arrest in APL cells, and promote APL cell differentiation combined with decreased DDX5 expression and increased reactive oxygen species (ROS) production. Knockdown of DDX5 by siRNA also inhibited proliferation, promoted cell differentiation and enhanced ROS production in APL cells. However, the ROS inhibitor reversed the effects of 2F5 on DDX5 and ROS in APL cells. Thus, we conclude that DDX5-targeting 2F5 inhibits APL cell proliferation, and promotes cell differentiation via induction of ROS. 2F5 showed the therapeutic value of fully human monoclonal autoantibody in APL, which provides a novel and valid approach for treatment of relapse/refractory APL.

## Introduction

Acute promyelocytic leukemia (APL) is a subtype of acute myeloid leukemia (AML) characterized by specific biological and clinical features. APL is distinguished by t (15; 17) chromosomal translocation^1^, which causes the production of a fusion protein known as promyelocytic leukemia–retinoic acid receptor (PML-RARα)^2^. APL has been characterized by early onset of clinical signs, disseminated intravascular coagulation and poor response to chemotherapy. Though previously marked by high mortality, it is nowadays the most curable form of AML^3^. AML therapy is comprised of therapeutic agents that induce apoptosis or promote the differentiation of cancer cells. At present, APL is treated by all-trans retinoic acid (ATRA) in combination with arsenic trioxide (ATO) or by ATRA and chemotherapy^4–6^. However, the resistant to ATRA and ATO of relapse/refractory APL patients is recognized as a critical problem in clinical practice^7^. Therefore, finding alternative targeting drugs with low toxicity may bring prospective solution to the treatment of relapse/refractory APL.

It has been demonstrated that AML patients had a complex karyotype which is marked by aberration expression of dead-box helicases^8^. Dead-box helicase 5 (DDX5) is a member of this family. Experimental depletion of DDX5 inhibits proliferation of AML cells and induces apoptosis by promoting the production of ROS^9^. Similarly, DDX5 is required in T-cell acute lymphoblastic leukemia (T-ALL) pathogenesis, which is evidenced by the decreased survival rate and inhibited proliferation following depletion of DDX5^10^. All these findings indicated that DDX5 may be a potential drug target in the treatment of APL.

Herein, a DDX5-targeting fully human monoclonal autoantibody named after 2F5 was prepared. And then the application potential of 2F5 in the therapy of APL was assessed. Results showed that 2F5 not only markedly inhibited the proliferation of APL cells, but also promoted APL cell differentiation by increasing ROS production. Considering the nontoxicity of 2F5 in cell viability, this study could provide a basis for the potential use of 2F5 in relapse/refractory APL therapy.

## Materials and methods

### Ethics statement

Experiments involving human and animal samples were approved by the Research Ethics Review Committee of Hangzhou Normal University. Animal procedures performed in this work followed guidelines in accordance with the Regulations for the Administration of Affairs Concerning Experimental Animals. Written informed consents were obtained from all participants.

### The preparation of DDX5-targeting fully human monoclonal autoantibody

Monoclonal antibodies were generated with hybridoma technology. SPYMEG (MBL, Nagoya, Japan)^11,12^ was used as a fusion partner cell for generating human monoclonal antibody that recognizes DDX5 specifically. Peripheral blood mononuclear cells (PBMCs) were obtained from the blood sample of SLE patient, and then were fused with SPYMEG to yield hybridomas. The resulting hybridomas were screened for DDX5-specific antibody secretion and cloned by limiting dilution. One stable clone secreting anti-DDX5 human monoclonal autoantibody was obtained and named after 2F5. The specific binding and affinity between 2F5 and DDX5 (OriGene, Rockville, USA) was determined by Surface Plasmon Resonance (Biacore X100, GE, USA) (Fig. S1b).

### Cell lines and culture

The human APL cell lines (HL-60 and NB4), T-ALL cell lines (Jurkat and CEM-C7), and monocytic leukemia cell line (THP-1) were purchased from Jennio Biotechnology Co., Ltd (Guangzhou, Guangdong, CHN). Blood samples were obtained from healthy volunteer. Neutrophils were isolated with human neutrophil isolation Kit (STEMCELL, CA, USA). PBMCs and monocytes were extracted with isolation kit (Solarbio, Beijing, China). Cells were cultured in RPMI 1640 medium supplemented with 10% fetal bovine serum (Gibco, Waltham, MA, USA) at 37 °C in a humidified incubator with 5% CO_2_. Cells were cultured in culture medium (normal control), and were treated with 2F5 or IgG (negative control) with different concentrations (20, 40, and 80 μM) for 4, 8, 12, and 16 days. Every 4 days, the cultures were established by centrifugation and then the cell pellets were resuspended in fresh corresponding medium respectively.

### Animal treatment and pathological examination

The 8-week-old Balb/c male mice were obtained from the Center for Laboratory Animals, Hangzhou Normal University, and were randomly divided into two groups. Mice were treated with 12 μg/kg 2F5 by intravenous injection (*n* = 6). Control mice received the same volume of saline (*n* = 6). Mice were euthanized with inhalation of anesthesia 24 h after injection. Tissue samples were fixed, dehydrated, embedded in paraffin, and sectioned into 4-µm slices for subsequent H&E staining. Wright-Giesma staining was performed using a staining kit (Solarbio, Beijing, China).

### Cell proliferation assay

Cell Counting Kit-8 (CCK-8, Sigma Aldrich, St. Louis, MO, USA) was used to detect cell proliferation, according to the manufacturer’s protocol. Cells were divided into three groups including 2F5 treatment group, IgG control and normal control group, and then 1 × 10^4^ cells were seeded with 100 μL different treatment medium per well in 96-well plate. CCK-8 solution was added to each well, and incubated for 4 h in the incubator. The absorbance was measured at 450 nm using a microplate reader. The living cell number in each group was calculated by the formula from standard curve.

### Western blotting

Protein lysates (50 μg/lane) were resolved on appropriate SDS-PAGE gels and transferred to PVDF membrane (Millipore, Billerica, MA, USA). The membranes were blocked, incubated with the indicated primary antibodies at 4 °C overnight, and then with the appropriate horseradish peroxidase-conjugated secondary antibodies. Blots were quantified with Image J (National Institutes of Health, Bethesda, MD, USA). Primary antibodies from Abcam (Cambridge, USA) were used: DDX5 (#ab126730), cyclin A2 (#ab181591), CDK4 (#ab108357), cyclin D1 (#ab16663), PCNA (#ab92552). β-actin (#AC026, ABclonal, Wuhan, China) was used as an internal control.

### NBT reduction assay

Cells were harvested in number of 1 × 10^6^ and incubated with a 1:1 mixture of culture medium and PBS containing 2 mg/mL nitro blue tetrazolium (NBT) and 1 μg/mL phorbol 12-myristate 13-acetate (Sigma Aldrich, St. Louis, MO, USA) at 37 °C for 1 h. The reaction was stopped by adding 200 μL of solution of hydrochloric acid (1 M). The absorbance at 562 nm was measured by spectrophotometer (Bio-Rad, Hercules, CA, USA).

### Flow cytometry

APL cells were incubated with 2F5 with ladder concentrations and 1 mM ATRA (Sigma Aldrich, St. Louis, MO, USA) for 4, 8, 12, or 16 days. For flow cytometry analysis, 5 × 10^5^ cells were harvested and washed with PBS, and labeled for 20 min with PE-conjugated anti-human CD11b or CD14 antibody (BD Biosciences, San Jose, CA). After washing with PBS, cells were analyzed on flow cytometry (BD Biosciences, San Jose, CA).

### ROS detection

The intracellular ROS was determined by ROS assay kit (Solarbio, Beijing, China). In brief, cells in different treatment groups were harvested, rinsed with PBS and incubated with DCFH-DA at 37 °C for 30 min. Then the DCF fluorescence of the cells was detected by fluorospectrophotometer (Bio-Rad, Hercules, CA, USA) or flow cytometry at an excitation wavelength of 488 nm and an emission wavelength of 535 nm. Five micromolar of N-Acetyl-L-cysteine (NAC, APExBIO, Houston, USA) was added to culture medium for blocking the production of ROS. As a ROS positive control, cells were treated with 100 μM H_2_O_2_ for 48 h as described previously^13^.

### Cell cycle analysis

Cells (1 × 10^6^/mL) were harvested and were centrifuged. And then the cells were fixed with cold 70% ethanol overnight at 4 °C, and then were washed twice by cold PBS. Cells were resuspended with 0.5 mL PI/RNase staining solution. After gentle vortex, cells were incubated for 30 min in room temperature, protected against light. Then cell cycle was analyzed by flow cytometry. The cell cycle data was fitted and analyzed by ModFit LT (verity software house, USA).

### siRNA transfection

Small interference RNA (siRNA) targeting DDX5, siDDX5 #1, 5′-GGUGCAGCAAGUAGCUGCUGAAUAU-3′; siDDX5 #2, 5′-GGAAUCUUGAUGAGCUGCCUAAAUU-3′; and its negative control scrambled siRNA were synthesized from Sangon Biotech (Shanghai, China) Co., Ltd. siRNA targeting DDX5 and control siRNA were transfected to HL-60 and NB4 cells according to the manufacturer’s instructions. Briefly, cells were transfected with 50 nM siRNA in serum-free and antibiotics-free DMEM containing 5 µL of Lipofectamine 3000 (Invitrogen, Carlsbad, CA, USA). The medium was changed 6 h later with normal growth medium supplemented with FBS. After 96 h of transfection cells were harvested.

### Surface plasmon resonance (SPR)

SPR is used to generate affinity information on specific interactions between 2F5 and DDX5 (Biacore X100, GE, USA). An anti-IgG antibody was firstly covalently immobilized onto a CM5 chip. Then 25 μM 2F5 were captured noncovalently onto the surface via their Fc region providing an optimal analyte-binding orientation. The resulting complex was stabilized by crosslinking with EDC/NHS to avoid baseline drift during measurement and regeneration. Finally, the interaction between 2F5 and DDX5 was monitored. PBS was used as running buffer in the binding assay. DDX5 (100 nM) were injected at a flow rate of 50 µL/min. After association and dissociation of the analyte, a regeneration step was performed in order to remove the remaining bound analyte.

### Statistical analysis

Data are presented as mean ± standard error of the mean (SEM), and all the experiments were repeated at least three times. Statistical analysis was performed using SPSS 13.0. One-way analysis of variance (ANOVA) was used to compare the differences between control and treatment groups. *p* < 0.05 was considered statistically significant.

## Results

### APL cell proliferation inhibited by monoclonal autoantibody 2F5

**Fig. 1:**
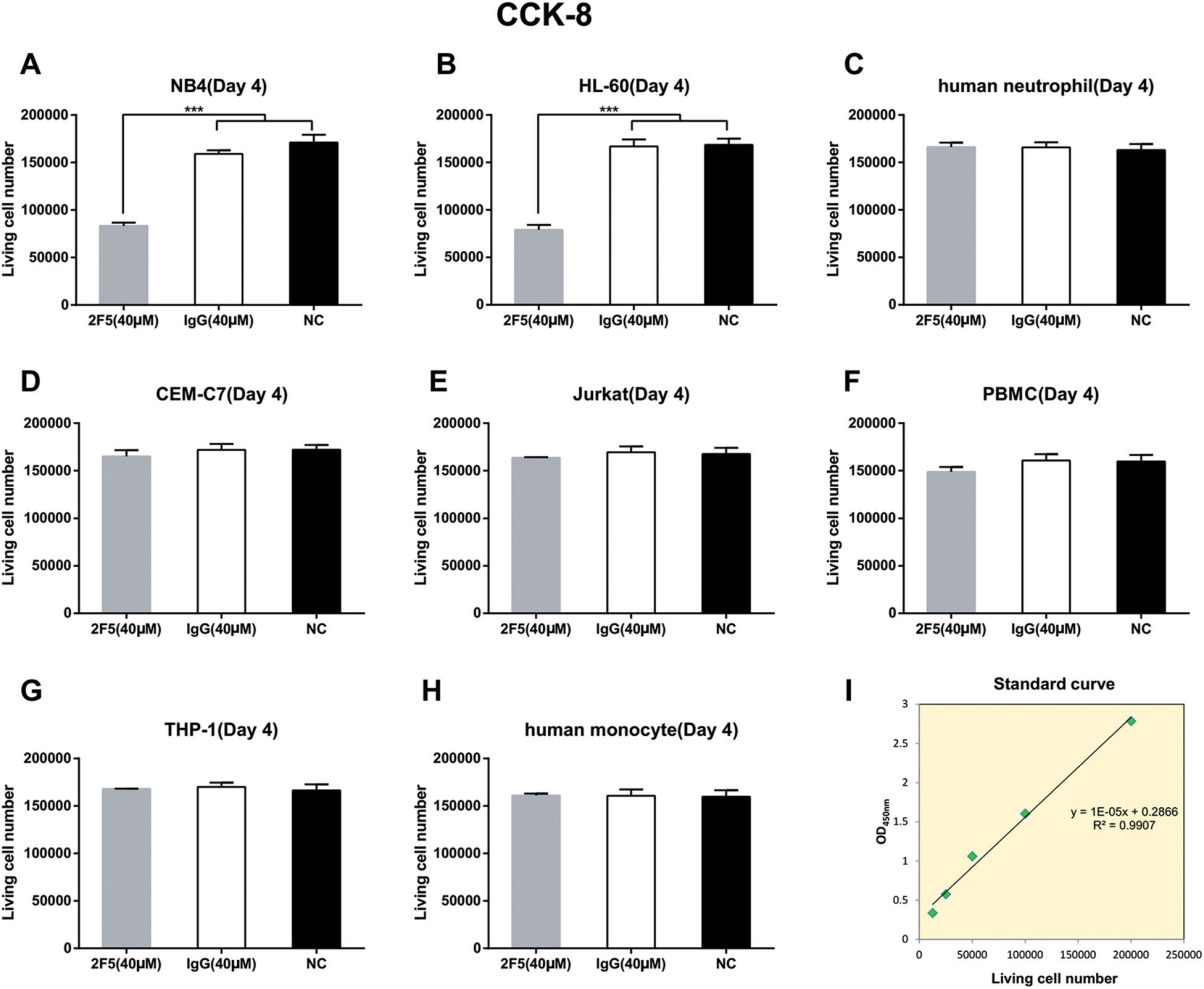
2F5 inhibits NB4 and HL-60 cell proliferation. CCK-8 assay was used to detect the living cell numbers of five AML cell lines and three corresponding healthy control cells 4 days after 40 μM 2F5 treatment. **a** 2F5 treatment significantly decreased NB4 living cell number. ***p < 0.001. **b** 2F5 treatment significantly decreased HL-60 living cell number. ***p < 0.001. **c** 2F5 had no effect on cell proliferation of human neutrophil. **d** 2F5 had no effect on cell proliferation of CEM-C7. **e** 2F5 had no effect on cell proliferation of Jurkat. **f** 2F5 had no effect on cell proliferation of PBMC. **g** 2F5 had no effect on cell proliferation of THP-1. **h** 2F5 had no effect on cell proliferation of healthy monocyte. **i** Standard curve was made to calculate living cell number in each group.

The effect of 2F5 on cell proliferation of different leukemia cell lines was detected by CCK-8 assay. Five cell lines including NB4, HL-60, Jurkat, CEM-C7, and THP-1 cells were treated with 40 μM 2F5, respectively for 4 days. Human neutrophil, PBMC, and monocyte were used as healthy controls. The results of CCK-8 assay indicated that 2F5 could significantly inhibit cell proliferation of NB4 and HL-60 cells compared with nontargeting IgG and normal control group (Fig. 1a, b, ***p < 0.001). However, 2F5 had no effect on cell proliferation of other three leukemia cell lines as well as the healthy controls (Fig. 1c–h). Cyclin D1 and PCNA expression levels in NB4 and HL-60 cells were analyzed for 4 days after 2F5 treatment with three different concentrations (20, 40, and 80 μM). Compared with both nontargeting IgG and normal control group, 2F5 induced a significant dose-dependent decrease of cyclin D1 and PCNA expression levels in NB4 and HL-60 cells (Fig. S2).

### Expression of DDX5 was elevated in APL cell lines

**Fig. 2:**
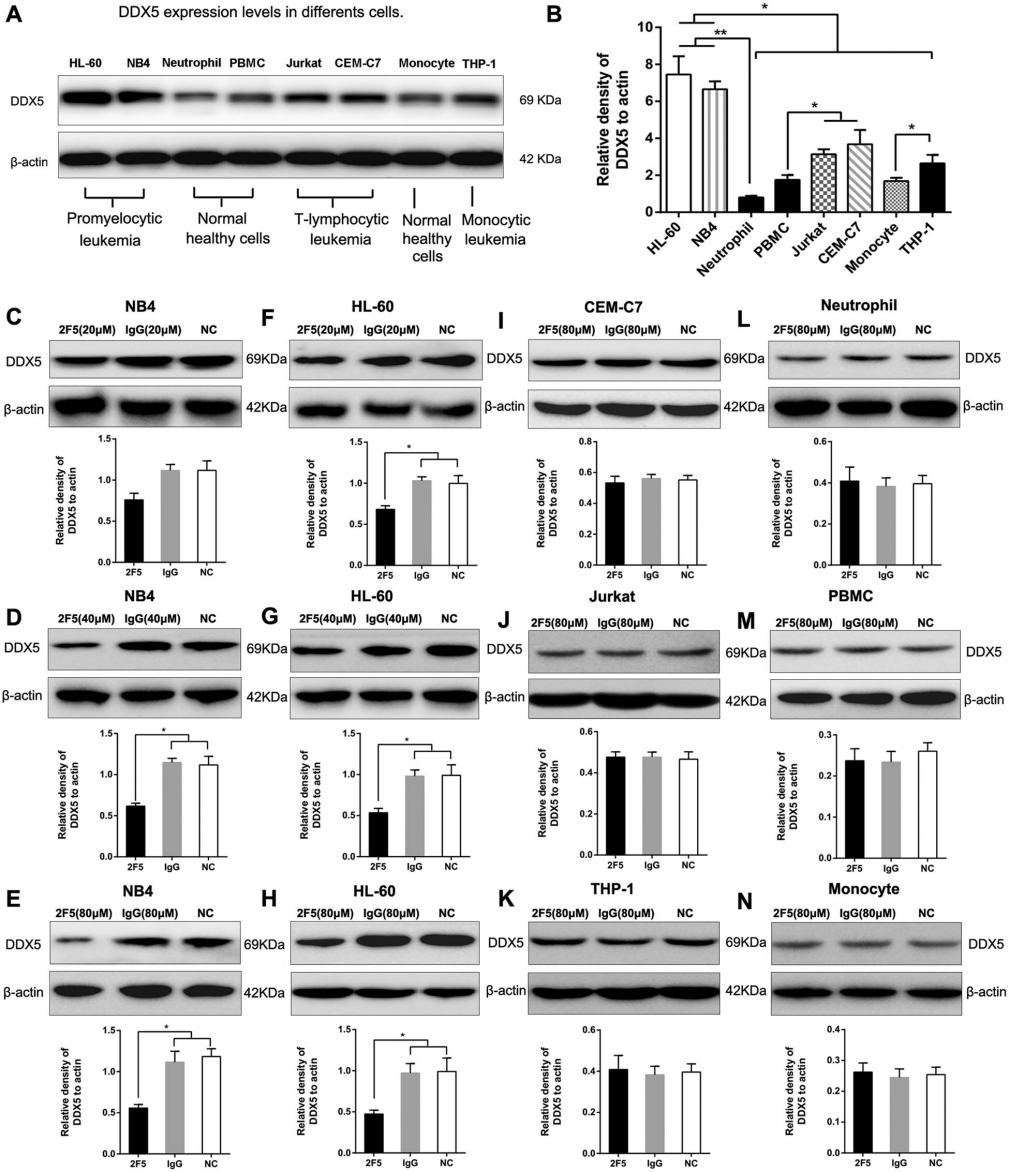
The effect of 2F5 on DDX5 expression levels in different leukemia cell lines. **a** Western blotting was performed to analyze the basal expression level in five leukemia cell lines and three corresponding healthy control cells. β-actin was used as an internal control. **b** DDX5 levels in NB4 and HL-60 cells are much higher than other six types of cells. *p < 0.05. DDX5 levels in three healthy control cells (neutrophil, PBMC and monocyte) are lower than in promyelocytic lymphoblastic and monocytic leukemia cells respectively. *p < 0.05. **p < 0.01. **c**–**e** DDX5 expression levels were detected by western blotting in NB4 cells 4 days after 20, 40, and 80 μM 2F5 treatment. *p < 0.05. **f**–**h** DDX5 expression levels were detected by western blotting in HL-60 cells 4 days after 20, 40, and 80 μM 2F5 treatment. *p < 0.05. **i** 80 μM 2F5 had no effect on DDX5 expression levels in CEM-C7 cells. **j** 80 μM 2F5 had no effect on DDX5 expression levels in Jurkat cells. **k** 80 μM 2F5 had no effect on DDX5 expression levels in THP-1 cells. **l** 80 μM 2F5 had no effect on DDX5 expression levels in neutrophils. **m** 80 μM 2F5 had no effect on DDX5 expression levels in PBMCs. **n** 80 μM 2F5 had no effect on DDX5 expression levels in monocytes.

The expression of DDX5 was detected in five different leukemia cell lines, and corresponding healthy control cells. As shown in Fig. 2, DDX5 expression levels in NB4 and HL-60 cells were much higher than the other six cell lines (*p < 0.05, Fig. 2a, b). Additionally, DDX5 levels in normal neutrophils, PBMCs and in monocytes were significantly lower than in APL (NB4 and HL-60), T-ALL (Jurkat and CEM-C7), and in monocyte leukemia (THP-1) cell lines (*p < 0.05, **p < 0.01, Fig. 2b).

### Downregulated DDX5 expression induced by 2F5 in APL cells

Five types of different leukemia cells were treated with 2F5. After 4 days, DDX5 expression was detected by western blotting. Results indicated that DDX5 expression were significantly suppressed in NB4 cells by 2F5 (*p < 0.05, Fig. 2d, e), as well as in HL-60 cells (*p < 0.05, Fig. 2f–h). However, there was no significant difference of DDX5 expression in CEM-C7, Jurkat, THP-1, healthy neutrophil, PBMC, and monocyte treated with 2F5, compared with IgG or normal control (Fig. 2i–n).

### G0/G1 phase arrest induced by 2F5 in APL cells

**Fig. 3:**
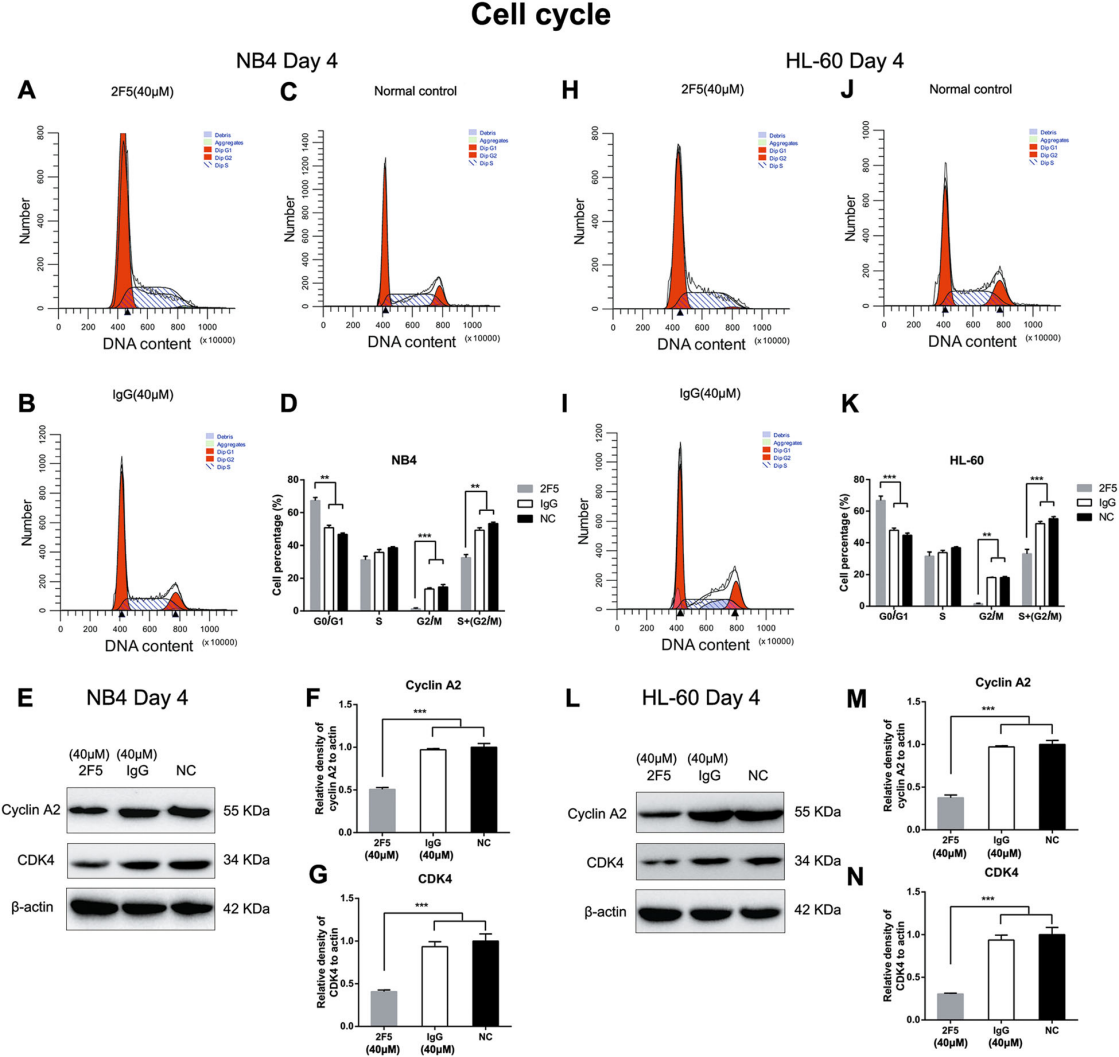
2F5 induces G0/G1 phase arrest in NB4 and HL-60 cells. **a**–**c** Cycle analysis was detected by flow cytometry in NB4 cells treated with 40 μM 2F5, 40 μM nontargeting IgG, and in untreated NB4 cells. **d** Percentage of NB4 cells in G0/G1 phase was higher than IgG and normal control. ***p < 0.001. However, percentages of 2F5-treated NB4 cells in G2/M and G2/M plus S phase were lower than IgG and normal control. **p < 0.01, ***p < 0.001. **e**–**g** Cell cycle related protein cyclin A2 and CDK4 expression levels were detected by western blotting in NB4 cells 4 days after 40 μM 2F5 treatment, 40 μM non-targeting IgG treatment, and in untreated NB4 cells. 2F5 significantly decreased cyclin A2 and CDK4 expression levels in NB4 cells. ***p < 0.001. **h**–**i** Flow cytometry was used for cell cycle analysis in HL-60 cells 4 days after 40 μM 2F5 treatment, 40 μM non-targeting IgG treatment, and in untreated HL-60 cells. **k** Percentage of HL-60 cells in G0/G1 phase was higher than IgG and normal control. **p < 0.01. However, percentages of 2F5-treated HL-60 cells in G2/M and G2/M plus S phase were lower than IgG and normal control. **p < 0.01. ***p < 0.001. **l**–**n** 2F5 significantly decreased cyclin A2 and CDK4 expression levels in HL-60 cells compared with IgG and normal control. ***p < 0.001.

The cell cycle distribution was analyzed by flow cytometry and western blotting in NB4 and HL-60 cells at day 4 after 40 μM 2F5 treatment. A significant increase in the G0/G1 phase was detected both in NB4 (**p < 0.01, Fig. 3a–d) and HL-60 cells (***p < 0.001, Fig. 3h–k). Meanwhile, 2F5 significantly decreased the percentages of G2/M and G2/M plus S phase in NB4 and HL-60 cells (**p < 0.01, ***p < 0.001, Fig. 3d, k). Consistently, cell cycle related protein Cyclin A2 and CDK4 expression levels were also found dramatically decreased in NB4 (***p < 0.001, Fig. 3e–g) and HL-60 cells (***p < 0.001, Fig. 3l–n) after 2F5 treatment.

### APL cells differentiation induced by 2F5 had no harmful effects on normal neutrophils and tissues

To examine whether 2F5 contributed to APL cell differentiation, Wright–Giemsa staining and NBT reduction assay were performed. Morphological features of cell differentiation were observed in 2F5-treated HL-60 cells and ATRA positive control group, such as a lower nucleocytoplasmic ratio and chromatin condensation (Fig. S3). Specially, 2F5-treated HL-60 cells showed granulocytic differentiation morphological features, including shrinkage nucleus, lobulated nucleus, and grayish cytoplasm (Fig. S3e, f).

Furthermore, NBT reduction assay was performed to detect cell differentiation. The result of NBT reduction assay showed that in NB4 cells, 40 μM 2F5 induce a significant increase of NBT reduction activity at day 4, 8, and 12 after 2F5 treatment (*p < 0.05, **p < 0.01, ***p < 0.001, Fig. S4a–c). Two curves showed that 2F5 played a time-dependent increasing effect on NBT reduction in NB4 and HL-60 cells (Fig. S4d, h).

**Fig. 4:**
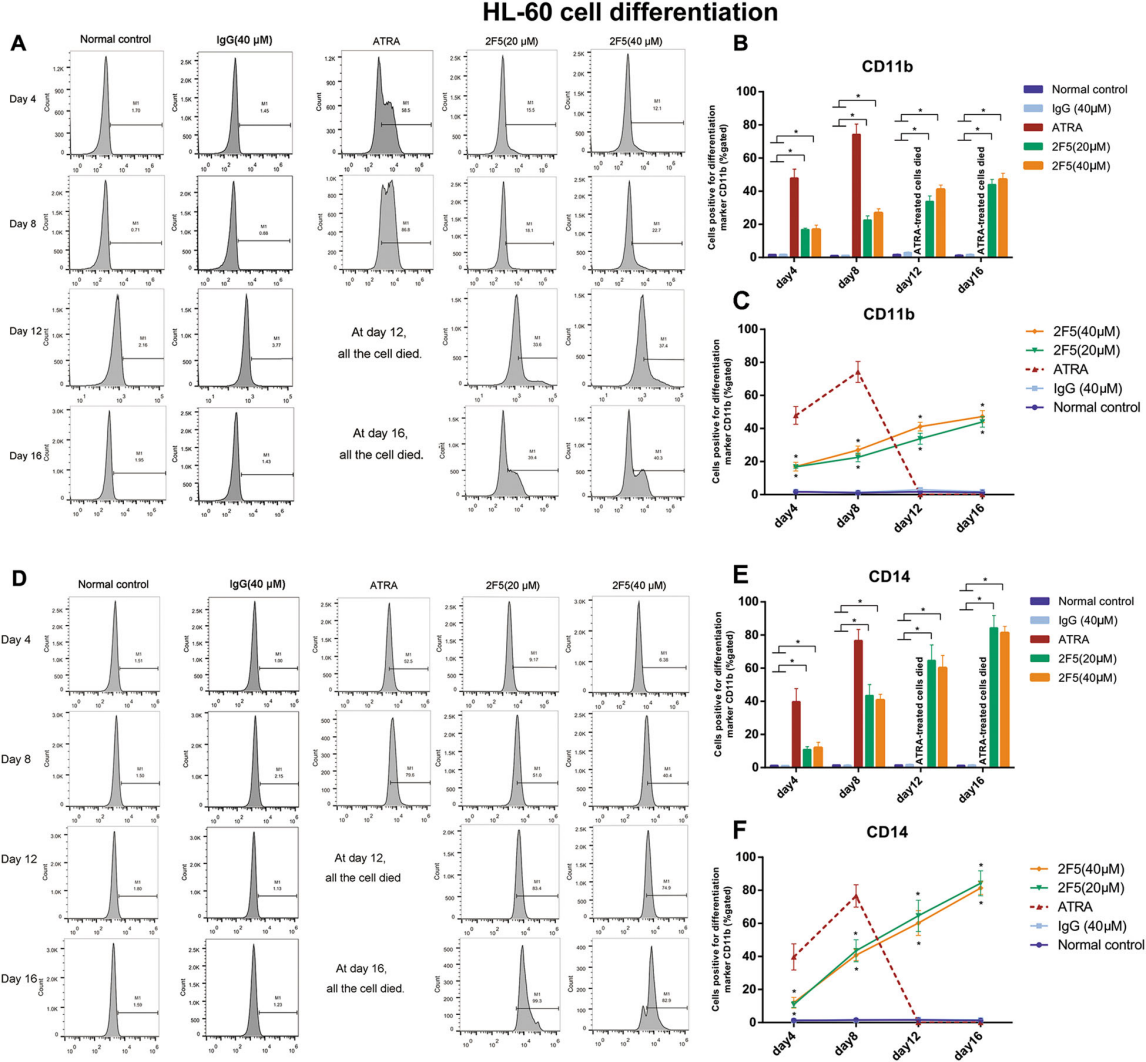
2F5 induces differentiation of HL-60 cells. Expression of the differentiation marker CD11b and CD14 were analyzed by flow cytometry in HL-60 cells treated with 2F5. Cells were obtained from the following treatment groups: untreated normal control, IgG control, ATRA treatment (positive control), 20 μM and 40 μM 2F5 treatment. **a**–**c** Percentages of CD11b-positive HL-60 cells in different groups at different time points were showed by straight peak map, column diagram and curves. At four time points, CD11b-positive cell ratio was significantly increased by 20 μM and 40 μM 2F5 treatment. *p < 0.05. **d**–**f** Percentages of CD14-positive HL-60 cells were detected by flow cytometry. At four time points, CD14-positive percentage was significantly increased by 20 μM and 40 μM 2F5 treatment. *p < 0.05.

**Fig. 5:**
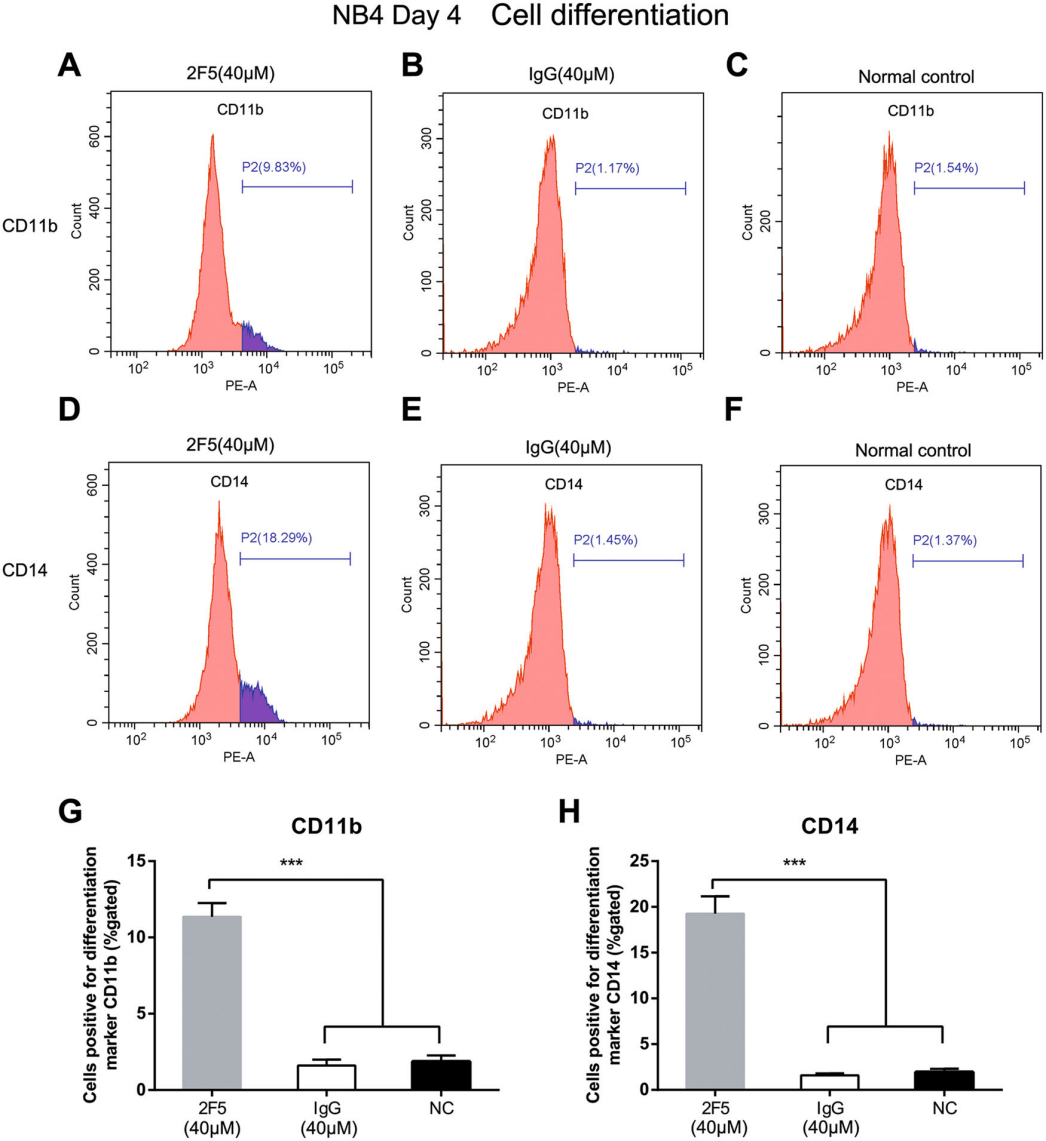
2F5 induces differentiation of NB4 cells. Expression of CD11b and CD14 in NB4 cells were analyzed by flow cytometry 4 days after 2F5 treatment. **a**–**c** Percentage of CD11b-positive NB4 cells in 40 μM 2F5 treatment group, 40 μM IgG treatment group and in untreated normal group. **d**–**f** Percentage of CD14-positive NB4 cells in 40 μM 2F5 treatment group, 40 μM IgG treatment group and in untreated normal group. **g** Percentage of CD11b-positive NB4 cells in 2F5 treatment group was higher than in IgG control and normal control group. ***p < 0.001. **h** Percentage of CD14-positive NB4 cells in 2F5 treatment group was higher than in IgG control and normal control group. ***p < 0.001.

Cells were found dead 8 days after ATRA treatment (Fig. S4 and Fig. 4), suggesting critical toxic effect of ATRA on cells. The population of CD11b-positive and CD14-positive HL-60 cells significantly increased following 2F5 or ATRA treatment compared with control (*p < 0.05, Fig. 4b, e). Interestingly, even if 2F5 exposure was stopped at day 12, the increasing trend of CD11b-positive and CD14-positive cells were kept to day 16. At day 16, CD14-positive HL-60 cells of 2F5 treatment group was higher than that in ATRA positive control group at day 8 (Fig. 4e). The percentage of CD11b-positive and CD14-positive cells persistently increased from day 4 to day 16 (Fig. 4c, f). Flow cytometry result showed that 2F5 promoted the cell differentiation with a time-dependent instead of dose-independent manner. In addition, similar results were acquired in NB4 cells that both CD11b-positive and CD14-positive cells were significantly increased by 2F5 treatment (***p < 0.001, Fig. 5).

In order to confirm the biological safety of 2F5, neutrophils were extracted from the blood of healthy volunteer. Then cell proliferation was detected by CCK-8 assay. The results indicated that there was no significant difference of cell proliferation between 2F5-treatment, normal control, and IgG control (Fig. S3m). Morphological staining was also performed on mice tissues. In animal model, no significant pathological changes were found in brain, kidney, or liver of mice with 2F5 intravenous injection (Fig. S3i–k).

### ROS production induced by 2F5 in NB4 and HL-60 cells

**Fig. 6:**
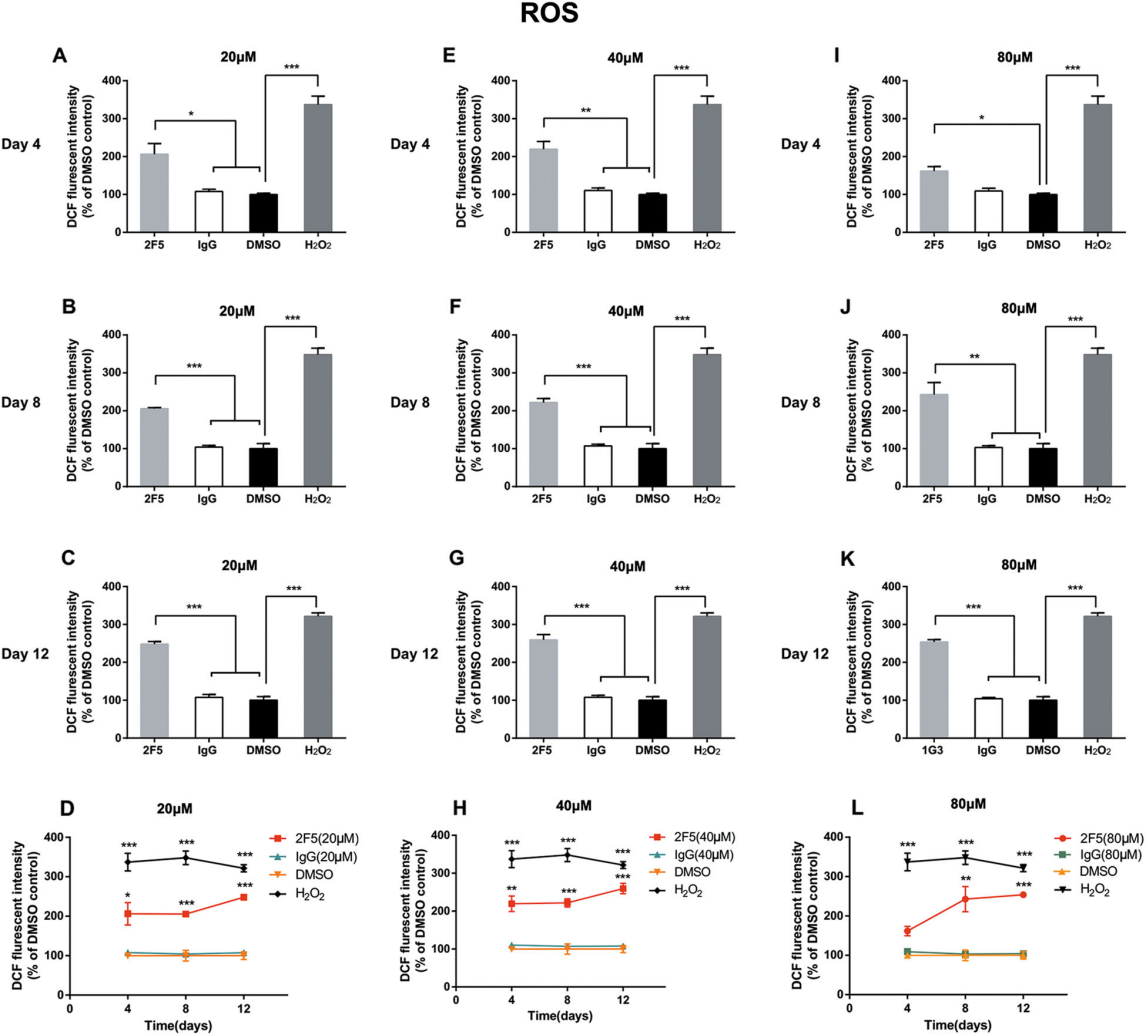
2F5 increases ROS production in HL-60 cells. HL-60 cells were divided into four treatment groups: 2F5 treatment group, IgG control group, DMSO control group and 100 μM H_2_O_2_ positive control group. The ROS production was determined by using an oxidant-sensing probe DCFH-DA. **a**–**d** 20 μM 2F5 significantly increased ROS production in HL-60 cells at day 4, 8, and 12 after 2F5 treatment. *p < 0.05. ***p < 0.001. **e**–**h** 40 μM 2F5 significantly increased ROS production in HL-60 cells at day 4, 8, and 12 after 2F5 treatment. **p < 0.01. ***p < 0.001. **i**–**l** 80 μM 2F5 significantly increased ROS production in HL-60 cells at day 4, 8, and 12 after 2F5 treatment. *p < 0.05. **p < 0.01. ***p < 0.001.

It has been reported that increased ROS levels can induce AML cell differentiation^14^, and ROS production is also critical for macrophage differentiation^15^. Hence, we sought to determine ROS level in APL cells treated with 2F5 by CM-H_2_DCFDA ROS detection assay. Compared with IgG control, 20 μM and 40 μM 2F5 induced a significant increase of ROS production at day 4, 8, and 12 (*p < 0.05, **p < 0.01, ***p < 0.001, Fig. 6a–h). The 80 μM 2F5 significantly increased ROS production at day 8 and day 12 (**p < 0.01, ***p < 0.001, Fig. 6j–l) except the day 4 compared with IgG control (Fig. 6i). The curve diagraph showed that the effect of 2F5 on ROS production in HL-60 cells was enhanced with the prolongation of treatment time, but not dose-dependent (Fig. 6d, h, l). These findings were consistent with the results of NBT assay (Fig. S4) and flow cytometry (Fig. 4). Furthermore, increased ROS production was also detected in NB4 cells treated with 2F5, but not in CEM-C7, Jurkat and THP-1 cells (**p < 0.01, Fig. S5). These results indicated that 2F5 may exert its role to promote leukemia cell differentiation by ROS production.

### ROS inhibitor blocked 2F5-induced cell differentiation

**Fig. 7:**
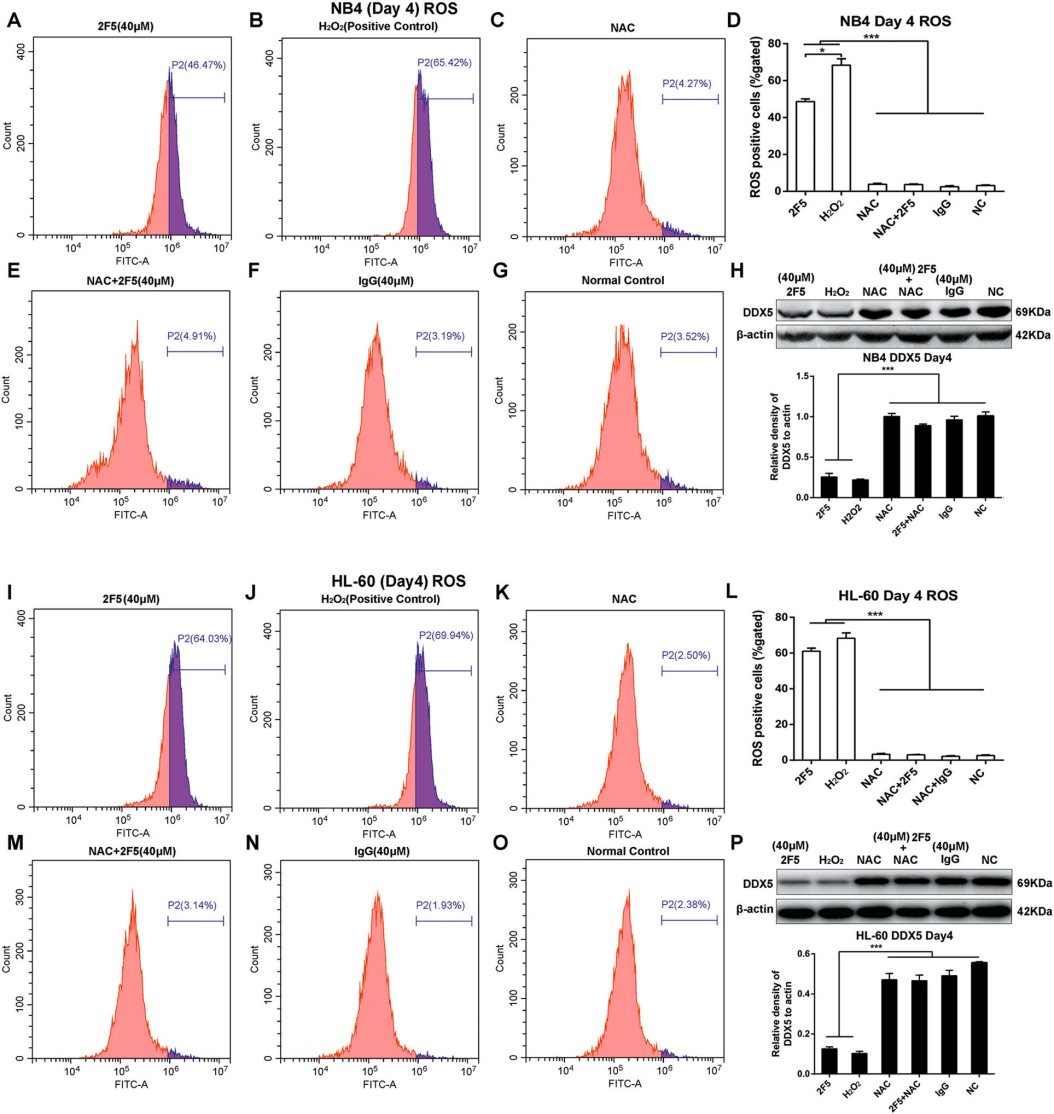
NAC could block the ROS promotion and DDX5 inhibition effects of 2F5 in NB4 and HL-60 cells. HL-60 and NB4 cells were divided into six treatment groups: 40 μM 2F5 treatment group, 100 μM H_2_O_2_ positive control group, 5 mM NAC treatment group, 5 mM NAC combined 40 μM 2F5 treatment group, 40 μM IgG treatment group, and untreated normal control group. The ROS production was analyzed by flow cytometry. **a**–**g** ROS positive cell percentage in NB4 cells treated with 2F5 combined NAC were much lower than cells treated with 2F5. ***p < 0.001. **i**–**o** ROS positive cell percentage in HL-60 cells treated with 2F5 combined NAC were much lower than cells treated with 2F5. ***p < 0.001. **h** 2F5 combined NAC reversed the inhibition effect of 2F5 on DDX5 expression level in NB4 cells. ***p < 0.001. **p** 2F5 combined NAC reversed the inhibition effect of 2F5 on DDX5 expression level in HL-60 cells. ***p < 0.001.

To determine whether ROS are required to maintain the promoting differentiation effect of 2F5, ROS inhibitor NAC was employed in NB4 and HL-60 cells, and then the ROS production, DDX5 expression and cell differentiation were analyzed. Flow cytometric detection of ROS showed that NAC could block the ROS promotion by 2F5 in NB4 and HL-60 cells (Fig. 7e, m). Compared with cells treated with 2F5 only, ROS production was significantly decreased in APL cells treated with NAC combined 2F5 (***p < 0.001, Fig. 7d, l). Moreover, DDX5 expression was significantly decreased in 2F5-treated NB4 and HL-60 cells, while increased combined with NAC treated group (***p < 0.001, Fig. 7h, p). These results suggested that the inhibition effect of 2F5 on DDX5 expression could be reversed by ROS inhibitor. In addition, flow cytometry detection of CD14 showed that compared with 2F5-treatment group, CD14-positive APL cells treated with 2F5 combined NAC were decreased significantly (Fig. S6). Collectively, these data indicated that the effect of 2F5 on promoting APL cell differentiation and inhibition of DDX5 expression is dependent on ROS production.

### The role of DDX5 silence on cell proliferation, ROS production, and cell differentiation

**Fig. 8:**
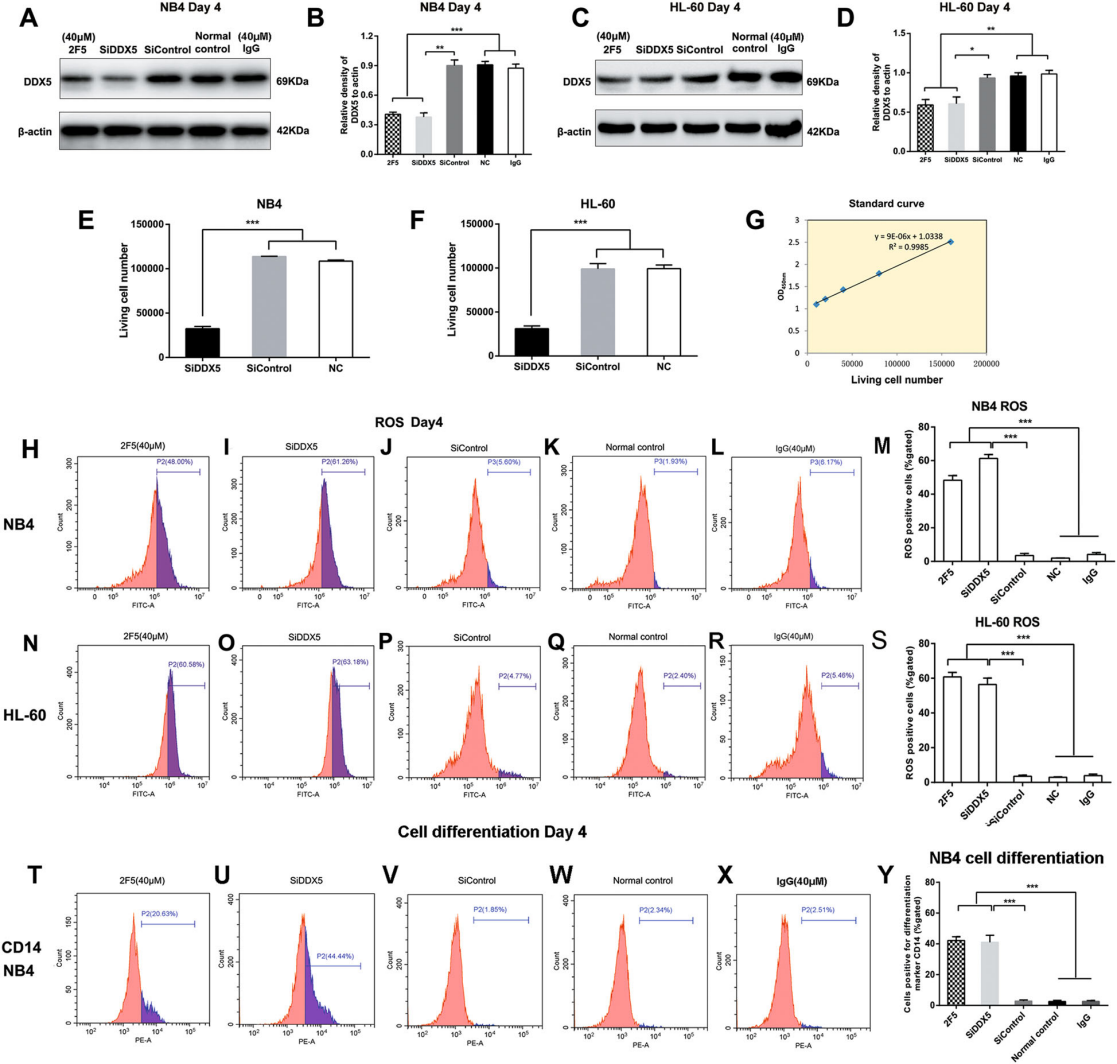
siDDX5 transfection decreases DDX5 expression level, inhibits cell proliferation, promotes ROS production, and promotes cell differentiation in NB4 and HL-60 cells. **a**–**d** HL-60 and NB4 cells were divided into five treatment groups: 40 μM 2F5 treatment group, siDDX5 transfection group, siControl transfection group, untreated normal control group, and isotypic IgG control group. **a**–**d** Both siDDX5 and 2F5 treatment could significantly reduce DDX5 expression levels in NB4 and HL-60 cells. *p < 0.05. **p < 0.01. ***p < 0.001. **e**–**g** Transfection of siDDX5 significantly reduced NB4 and HL-60 living cell numbers. ***p < 0.001. **h**–**m** Transfection of siDDX5 increased ROS production in NB4 cells. ***p < 0.001. **n**–**s** Transfection of siDDX5 increased ROS production in HL-60 cells. ***p < 0.001. **t**–**y** Transfection of siDDX5 induced an increase of CD14-positive cell percentage in NB4 cells. ***p < 0.001.

To determine whether DDX5 plays an essential role in regulating APL cell differentiation, siRNAs targeting DDX5 were transfected in NB4 and HL-60 cells. Western blotting analysis revealed significant decrease of DDX5 expression in siDDX5 transfected NB4 and HL-60 cells compared to control scramble siRNA respectively (*p < 0.05, **p < 0.01, Fig. 8a–d). Compared with siDDX5, 2F5 had similar ability on specific down-regulating DDX5 expression level, which revealed that 2F5 treatment is sufficient to inhibit DDX5 activity. siDDX5 significantly reduced the living cell number of NB4 and HL-60 cells (***p < 0.001, Fig. 8e, f). Moreover, similar to 2F5, siDDX5 also increased ROS production and CD14-posistive cell percentages in NB4 and HL-60 cells (***p < 0.001, Fig. 8m, s, y).

## Discussion

The therapeutic management of APL has considerably evolved during the past two decades. ATRA in combinatorial regimens with chemotherapy has provided high cure rates. But this approach shows significant toxicity including severe myelosuppression and occurrence of relapse/refractory leukemia^16^. In recent years, the advent of ATO and its use in combination with ATRA with or without chemotherapy has further improved patient outcome by maintaining high antileukemic efficacy. However, common complications such as differentiation syndrome and hepatic toxicity induced by ATO causes serious harm to patients^17^. Monitoring liver function and adjusting treatment regimens at any time during ATO therapy also bring problem and complexity to APL treatment^18^. Therefore, finding new management strategy without toxicity is dramatically valuable for targeting treatment of APL.

Autoantibody is immunoglobulin directed against an individual’s self-antigens, the function of which has been abundantly explored in SLE patients. In recent years, researchers have become more interested in the role and application of autoantibodies in treatment of human malignant tumors^19^. Numerous epidemiological studies showed that risk of some cancers in SLE were decreased^20^. For example, the risk of endometrial cancer in SLE patients is decreased more than 50%^21,22^, which implies that certain autoantibodies in people with SLE may prevent carcinogenesis.

Currently, existing monoclonal antibody drugs used for treating leukemia mostly targeted lymphocyte surface molecules, such as CD20, CD3, or CD19 and so on^23^. Beside those cell surface biomarkers, some transcriptional regulators have been reported to be aberrantly expressed in many cancers, and are linked to the regulation of many cancer-related pathways. Among those transcriptional regulators, the DDX5 plays a key role in the tumorigenesis, invasiveness, and metastasis^24^. It has been reported that AML is dependent on DDX5, and inhibition of DDX5 slows AML cell proliferation in vitro and AML progression in vivo, but is not toxic to cells from normal bone marrow^9^. These investigation conclusions provide an insight that DDX5-targeting monoclonal autoantibodies might be potential new agents for APL therapy.

DDX5 expression is closely correlated with the onset of organ differentiation^25^. The function of DDX5 is responsible for the expression of proteins that contribute to cell differentiation. Otherwise, studies showed that downregulation of DDX5 in gastric and lung cell lines abrogates proliferation^26,27^, which suggested that DDX5 could affect cancer cell proliferation. DDX5 is considered to be required for T-ALL pathogenesis since it regulates the notch signaling pathway which is required for the growth and survival of T-ALL cells. This is evidenced by Lin et al. that DDX5 depletion decreased survival rate and inhibited proliferation of KOPT-K1 and HPB-ALL cells^10^. Similar conclusion was also acquired in present study that both DDX5-targeting 2F5 and siRNA could inhibit APL cell proliferation by downregulation of DDX5. Moreover, 2F5 induced G0/G1 phase arrest in APL cells (Fig. 3). Interestingly, unlike the finding related to DDX5 by Lin et al., we found that DDX5-targeted 2F5 has no effect on the proliferation of T-ALL cell line (Jurkat and CEM-C7). Our results showed that the basal expression levels of DDX5 in different leukemia cell lines were also different, DDX5 basal level in APL cell lines were much higher than that in T-ALL cell line (Fig. 2). These findings indicated that the different basal level of DDX5 might determine the susceptibility of different leukemia subtypes to 2F5. Moreover, 2F5 makes no harmful effect on healthy neutrophils and tissues (Fig. S3). This provides evidence to safety of 2F5 in potential further application.

The differentiation promotion effect of 2F5 was evaluated by NBT reduction assay, morphological staining and differentiation biomarker analysis (Figs. 4, 5 and Figs. S3 and 4). CD11b and CD14 have been known as characteristic surface markers on mature granulocyte and mature monocyte, respectively^28^. Those two surface markers decide the differentiation trends of APL cells. In APL cells treated with 2F5, the percentage of CD14-positive cells was obviously higher than CD11b-positive cells, thus it is speculated that 2F5 could induce APL cell differentiation along the monocytic lineage more than myeloid lineage. Moreover, APL cells were found dead at day 8 after ATRA treatment, while APL cells were found still alive at day 16 after 2F5 treatment. It is suggested that 2F5 exerts its pro-differentiation effect on APL cell via other pathway that different from the pro-apoptosis mechanism of ATRA^29^. Of note, 2F5 exerted promotion effect on APL cell differentiation up to day 16, even though the treatment of 2F5 was stopped at day 12 (Fig. 4). It means that 2F5 exerts its pro-differentiation effect on APL cells for a long time.

Up to now, there is no monoclonal antibody medicine used clinically for APL therapy with differentiation inducing mechanism. A monoclonal antibody against CD44 used alongside ATRA and FICZ (6-Formylindolo (3, 2-b) carbazole) has been shown to increase cell apoptosis^30^. Another monoclonal antibody called HuM195 is used for APL targeted radiotherapy as a molecular chaperone agent by targeting CD33^31^. HuM195/rGel is a compound prepared by HuM195 coupling to plant toxin gelonin, which has been proven to be cytotoxic to HL-60 cells^32^. As a direct pro-differentiation monoclonal autoantibody, 2F5 has its own specific advantages and mechanism of action. In APL cells, 2F5 was found to inhibit DDX5 expression and promote ROS production significantly. Moreover, siRNA-targeted DDX5 played a similar role to 2F5 on APL cell proliferation, differentiation, and ROS production. However, the ROS inhibitor reversed the effect of 2F5 on DDX5 and ROS in APL cells. These findings indicated that 2F5 could promote APL cell differentiation by inhibition of DDX5 and activation of ROS.

It has been demonstrated that the decrease of DDX5 is the cause of ROS induction^9^. In addition, it has also been reported that NADPH oxidase-derived ROS plays a critical role in HL-60 cell monocytic differentiation^13^. ROS is involved in the HL-60 cell monocytic differentiation induced by isoliquiritigenin^13^. Combined with results in present study that ROS plays a dual role in promotion effect of APL cell differentiation. On one hand, DDX5 depletion-induced ROS production promotes APL cell differentiation. On the other hand, NAC-mediated ROS inhibition also blocks the decreasing effect of 2F5 on DDX5 and subsequent promotion effect of 2F5 on ROS and cell differentiation (Fig. S7). All these findings suggested that 2F5 promoted APL cell differentiation via targeted reducing DDX5-mediated ROS production. For the mechanisms by which autoantibodies penetrate living cells, researchers have suggested that Fc-receptor-mediated or some special cell membrane proteins mediate endocytosis to facilitate the cell penetration of autoantibodies^33^. However, endosomes prevent 2F5 to recognize and bind to its targeting protein DDX5^19^. For this reason, it is suggested that 2F5 decreased DDX5 level via endosome-independent pathway. It is speculated that 2F5 binds to extracellular nucleotide and transports to the cell plasma via equilibrative nucleoside transporter^34^, and then 2F5 neutralizes free DDX5 consequently (Fig. S7).

This study concluded that DDX5-targeting autoantibody 2F5 inhibited proliferation, induced a G0/G1 phase arrest, and promoted differentiation of APL cells by decreasing DDX5 and increasing ROS levels without cytotoxicity to healthy tissues and cells. This finding provides a novel and valid approach to solve the problem in therapy of relapse/refractory APL.

## Supplementary information

Figure S1

Figure S2

Figure S3

Figure S4

Figure S5

Figure S6

Figure S7

Supplementary figure legends
