# Supplementary figures and images for: DDX5-targeting fully human monoclonal autoantibody inhibits proliferation and promotes differentiation of acute promyelocytic leukemia cells by increasing ROS production
Source: Cell Death Dis. 2020 Jul 20;11(7):552. doi: 10.1038/s41419-020-02759-5 (PMC7371707; doi:10.1038/s41419-020-02759-5)

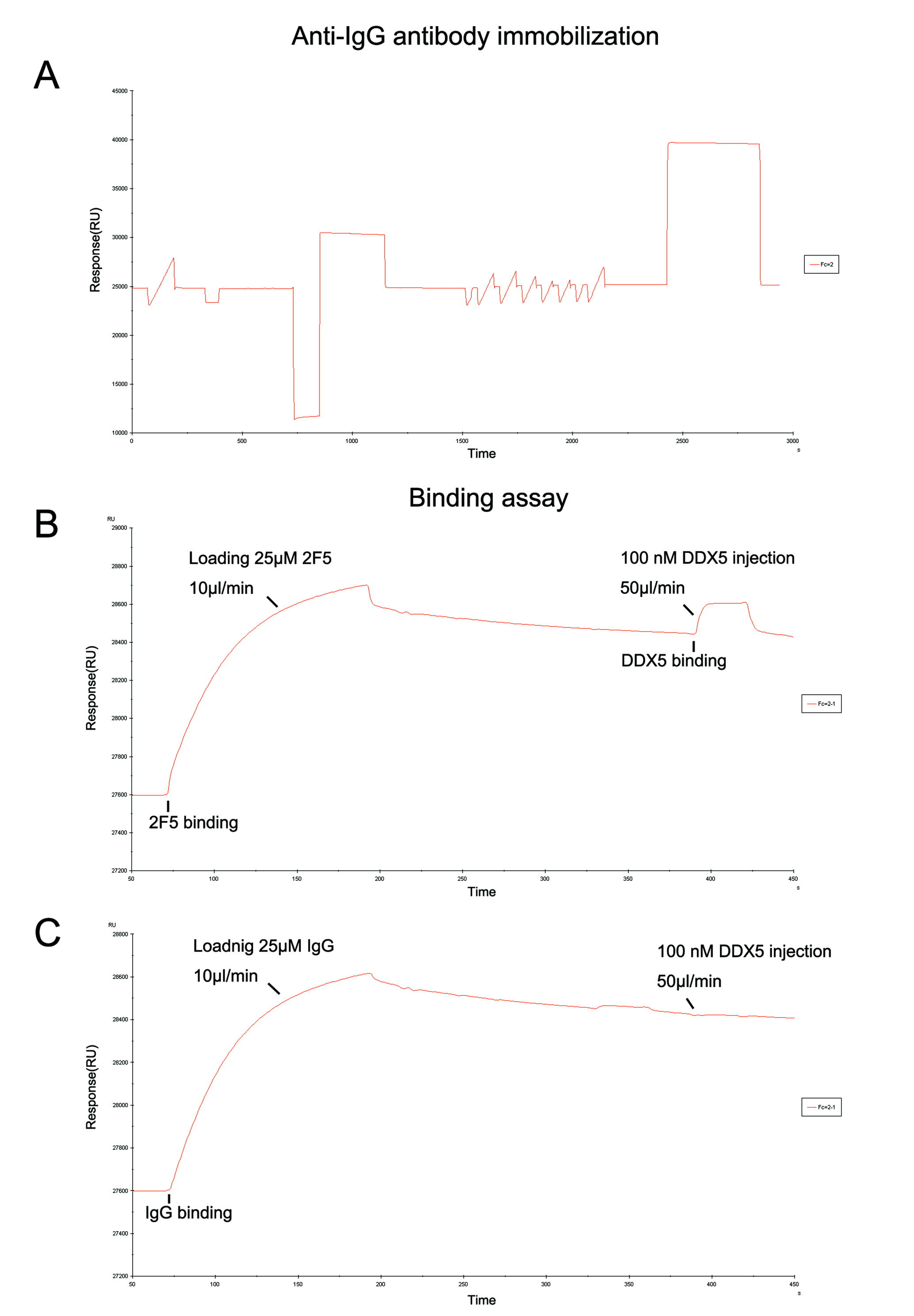

Supplement: Supplementary file 1 — Figure S1 [file 41419_2020_2759_MOESM1_ESM.tif]

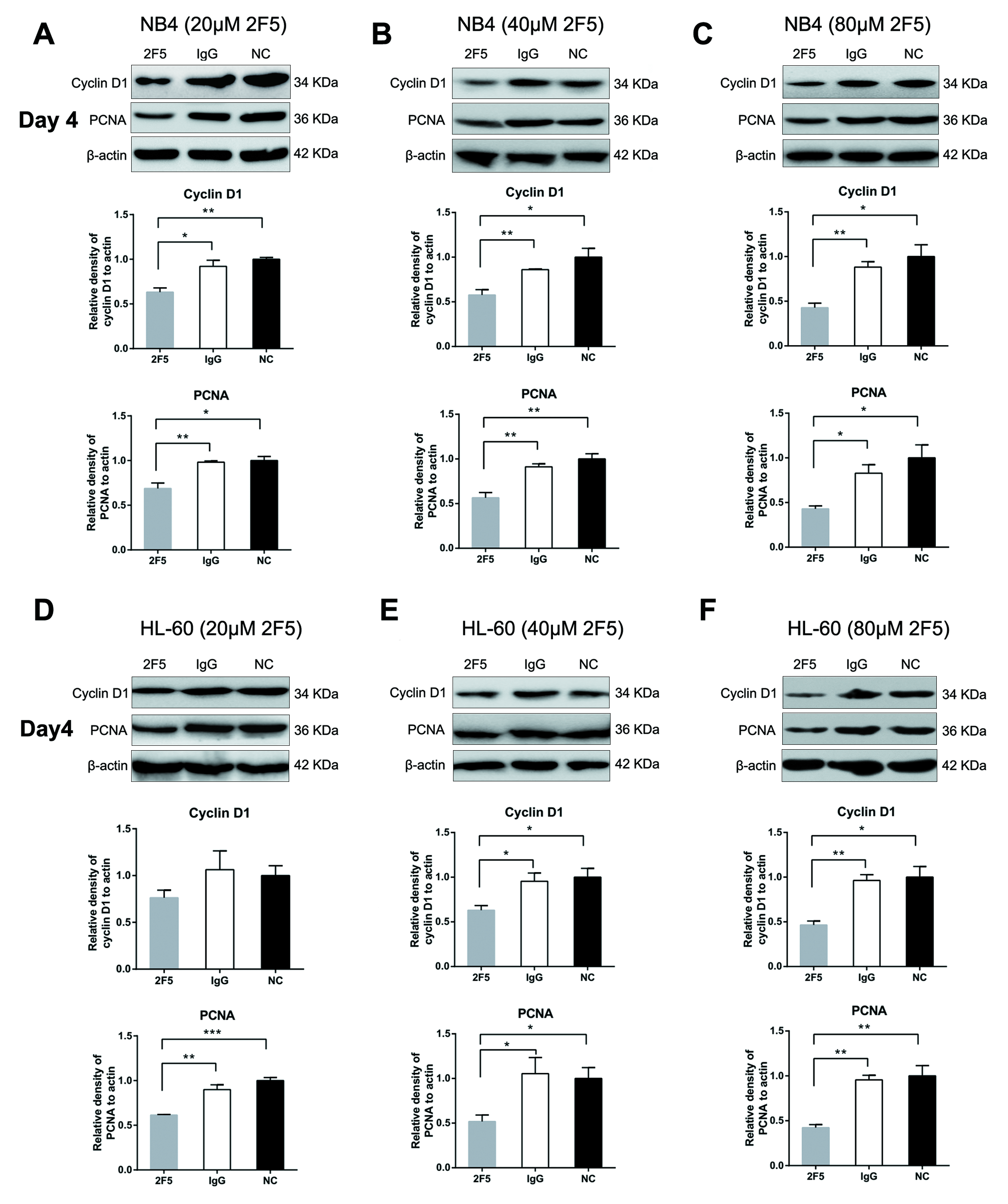

Supplement: Supplementary file 2 — Figure S2 [file 41419_2020_2759_MOESM2_ESM.tif]

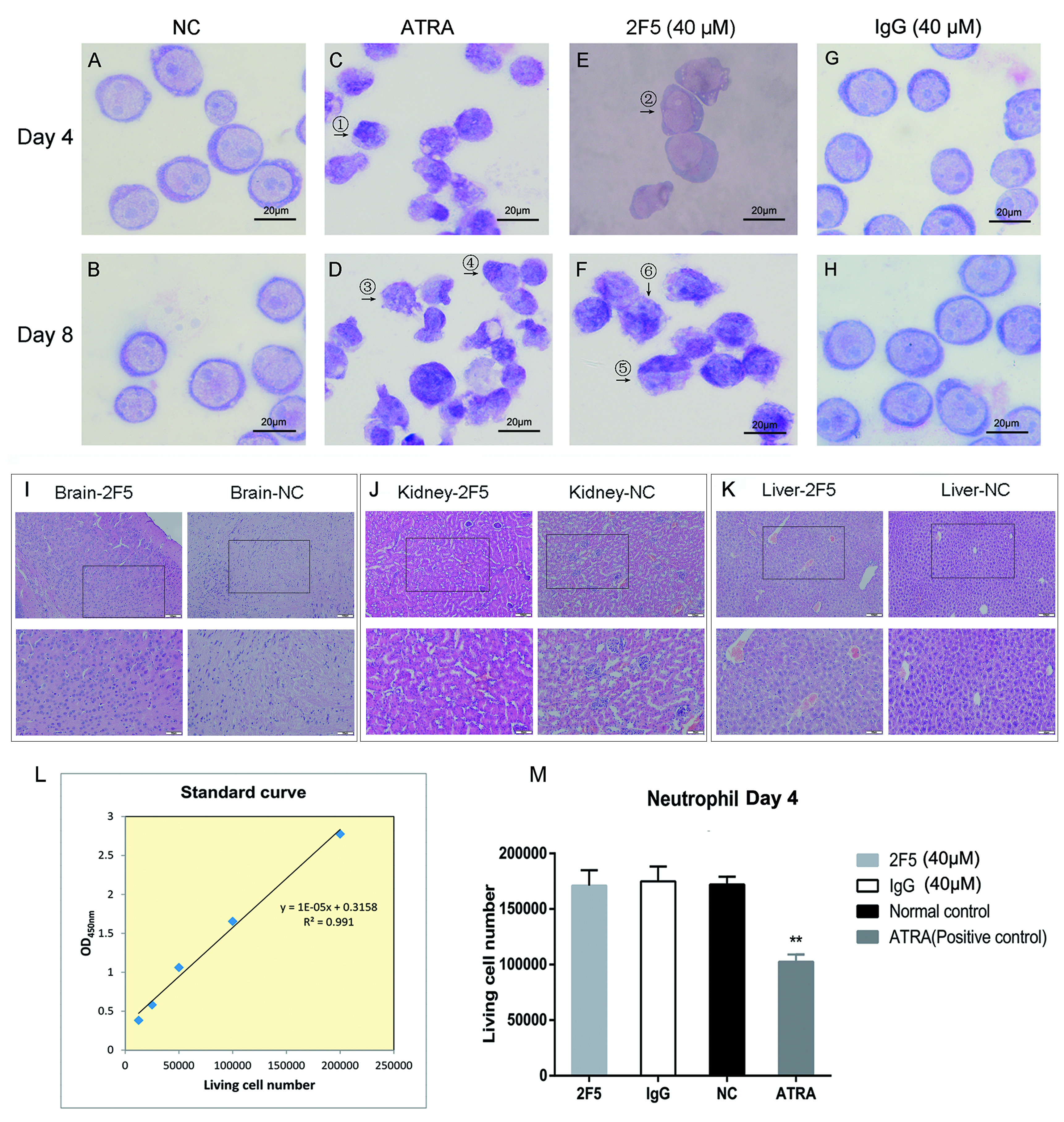

Supplement: Supplementary file 3 — Figure S3 [file 41419_2020_2759_MOESM3_ESM.tif]

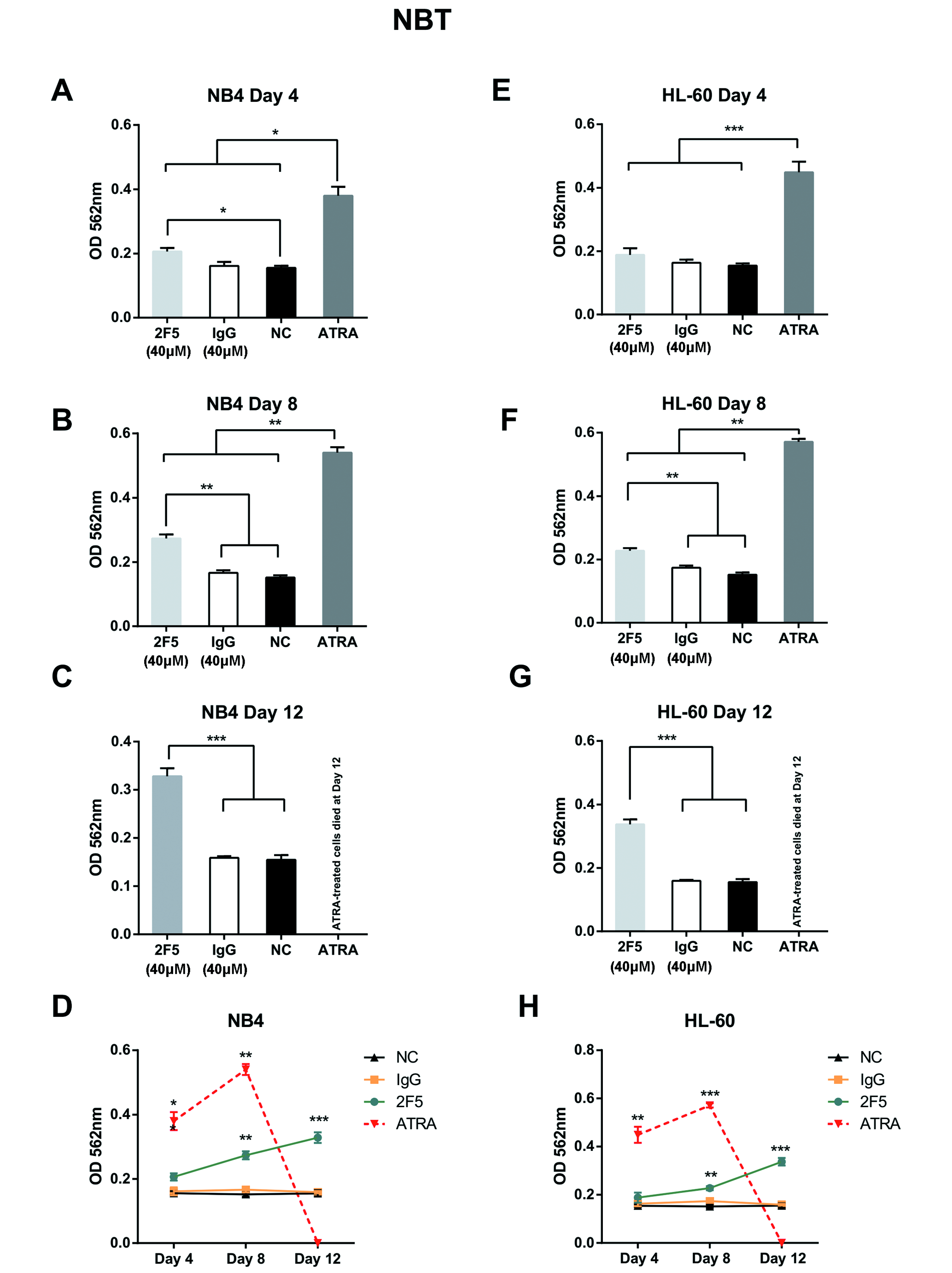

Supplement: Supplementary file 4 — Figure S4 [file 41419_2020_2759_MOESM4_ESM.tif]

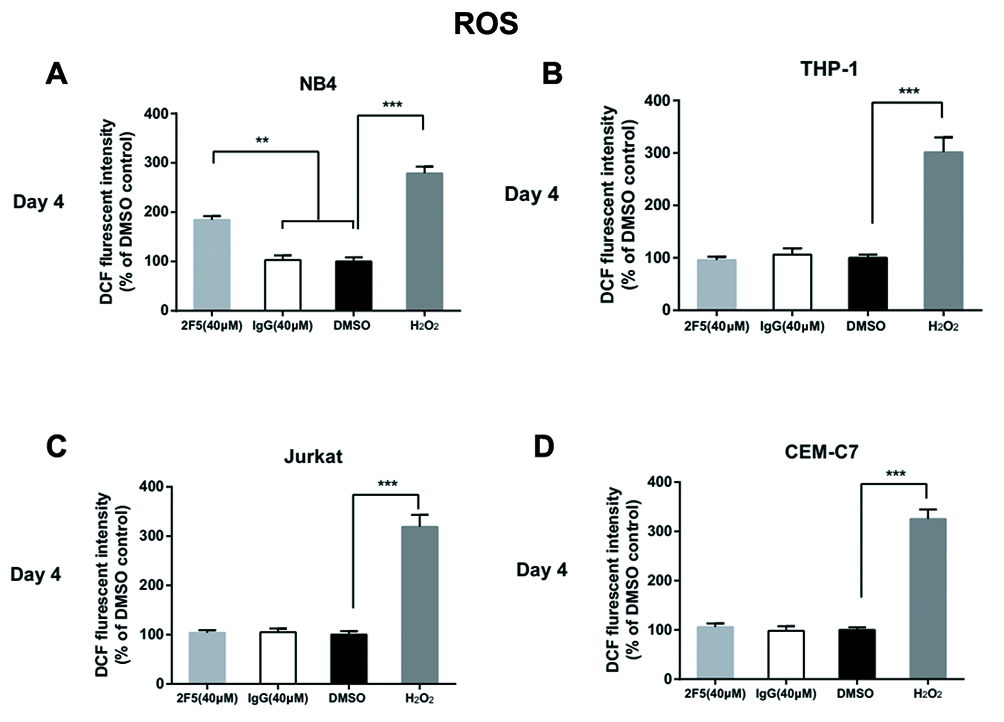

Supplement: Supplementary file 5 — Figure S5 [file 41419_2020_2759_MOESM5_ESM.tif]

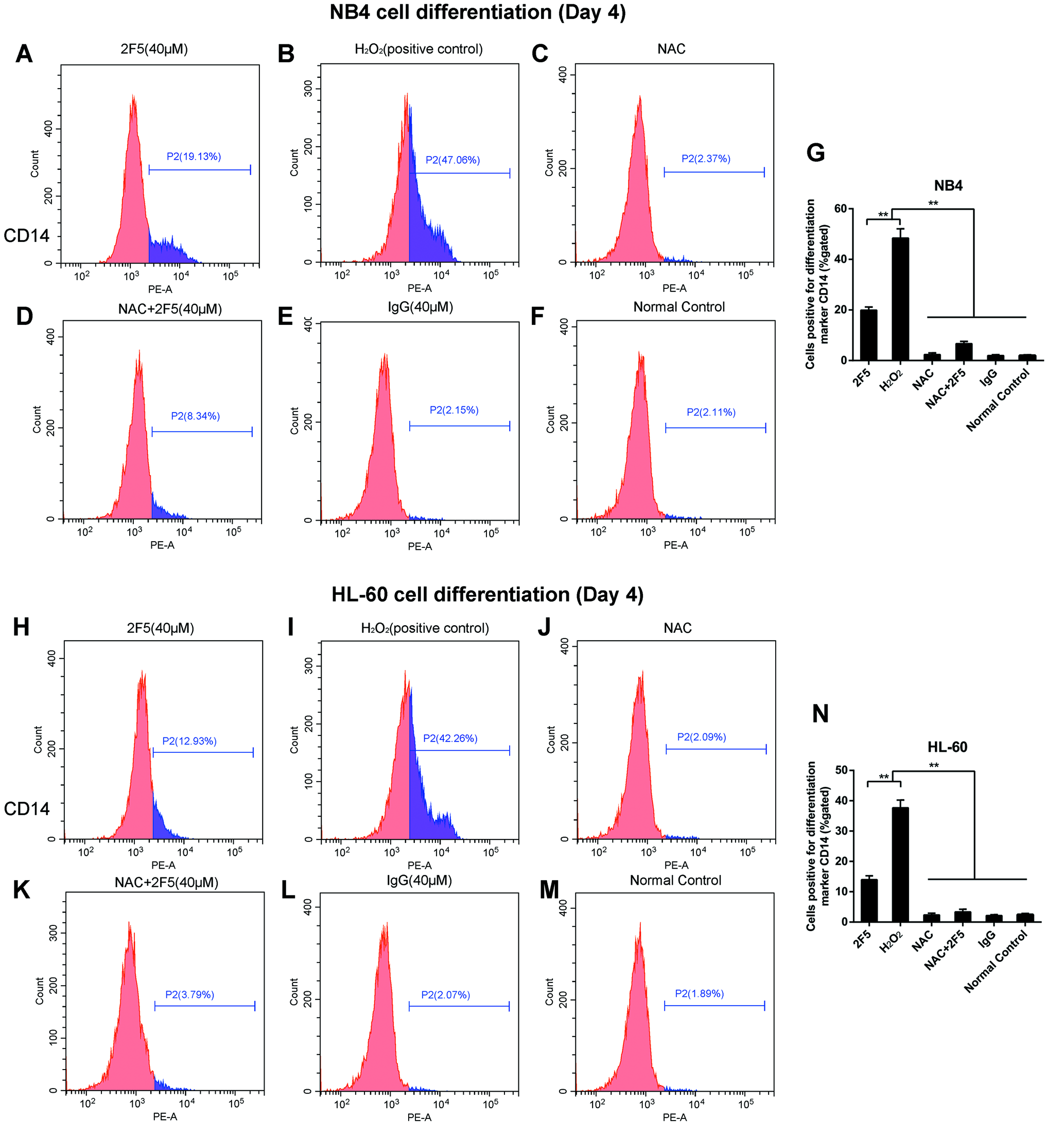

Supplement: Supplementary file 6 — Figure S6 [file 41419_2020_2759_MOESM6_ESM.tif]

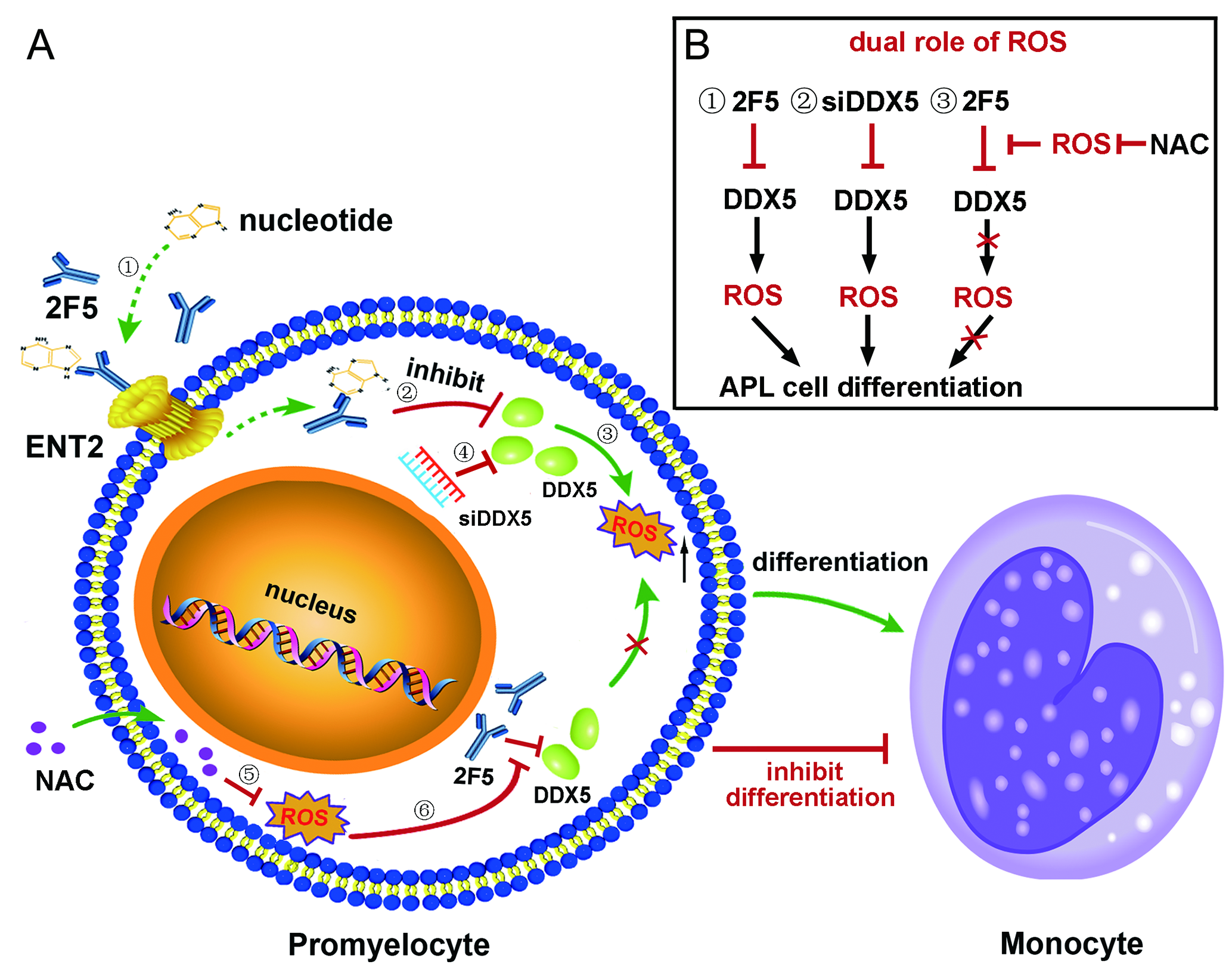

Supplement: Supplementary file 7 — Figure S7 [file 41419_2020_2759_MOESM7_ESM.tif]
