# Supplementary material for: DDX5-targeting fully human monoclonal autoantibody inhibits proliferation and promotes differentiation of acute promyelocytic leukemia cells by increasing ROS production
Source: Cell Death Dis. 2020 Jul 20;11(7):552. doi: 10.1038/s41419-020-02759-5 (PMC7371707; doi:10.1038/s41419-020-02759-5)
Supplement: Supplementary file 8 — Supplementary figure legends [file 41419_2020_2759_MOESM8_ESM.docx]

**Supplementary figure legends**

**Fig. S1 2F5 could specifically bind to its target protein DDX5.** The specific binding affinity between 2F5 and DDX5 was verified by surface plasmon resonance. **a** Anti-human IgG antibody was immobilized onto CM5 chip. **b** 2F5 and DDX5 protein were injected manually. The sensing diagraph showed that the combination of DDX5 and 2F5 had the characteristics of rapid combination and dissociation. **c** Non-targeting human IgG cannot bind to DDX5 specifically.

**Fig. S2 2F5 inhibited cyclin D1 and PCNA expression levels in NB4 and HL-60 cells.** Cell proliferation related proteins cyclin D1 and PCNA were detected in NB4 and HL-60 cells by western blotting four days after 2F5 treatment. **a-c** Compared with IgG control and untreated normal control, 20μM, 40μM and 80μM 2F5 significantly reduced cyclin D1 and PCNA expression levels in NB4 cells. **P*<0.05. ***P*<0.01. **d-f** Compared with IgG control and untreated normal control, 20μM, 40μM and 80μM 2F5 significantly reduced cyclin D1 and PCNA expression levels in HL-60 cells. **P*<0.05. ***P*<0.01. ****P*<0.001. β-actin was used as an internal control. Bands were measured using optical densitometry. In order to confirm whether the action of 2F5 has a concentration-dependent trend or not, the data in different concentration were normalized to normal control sample (defined as 1.0).

**Fig. S3 2F5 induced HL-60 cell differentiation without harmful effect on normal neutrophil and tissues. a-h** HL-60 cells were divided into four treatment groups: untreated control, 1mM ATRA treatment group (positive control), 40μM 2F5 treatment group, and non-targeting IgG control group. HL-60 cells were treated with 2F5 for four or eight days. HL-60 cells treated with ATRA or 2F5 displayed cell differentiation features, such as a lower nucleocytoplasmic ratio and chromatin condensation (shown by arrows ①, ②, ④ and ⑤). HL-60 cells treated with 2F5 showed granulocytic differentiation morphological features, including shrinkage nucleus, lobulated nucleus, and grayish cytoplasm (shown by arrow ⑥). However, some cells broke up and died in ATRA treatment group (shown by arrow ①). Original magnification was ×400 (objective lenses ×40) under a light microscope (Olympus, Tokyo, Japan), and images were captured using DP2-BSW software (Olympus). (Bar=20μm). **i-k** Morphological analysis was made by H&E staining in mice brain, kidney and liver after 2F5 intravenous injection. (Bar=100μm; Bar=50μm) **l and m** Neutrophil proliferation was analyzed by CCK-8 assay at day 4 after 40μM 2F5 treatment. 2F5 had no effect on proliferation of neutrophils.

**Fig. S4 Detection of NBT-reduction activity.** Cell differentiation of NB4 and HL-60 cells at several different time points (day 4, day 8, and day 12) was detected by NBT reduction assay after 2F5 treatment. **a-d** In NB4 cells, 40μM 2F5 induced a significant increase of NBT reduction activity at day 4, 8 and 12 after 2F5 treatment. **P*<0.05. ***P*<0.01. ****P*<0.001. **e-h** In HL-60 cells, 40μM 2F5 induced a significant increase of NBT reduction activity at day 8 and 12 after 2F5 treatment except at day 4. ***P*<0.01. ****P*<0.001.

**Fig. S5 Effect of 2F5 on ROS production in NB4, THP-1, Jurkat and CEM-C7 cell lines.** Cells were divided into four treatment groups: 40μM 2F5 treatment group, 40μM IgG control group, DMSO control group and 100μM H_2_O_2_ positive control group. Four days later, the ROS production was determined by using an oxidant-sensing probe DCFH-DA. **a** 2F5 treatment induced an increase of ROS production in NB4 cells. ***P*<0.01. **b** 2F5 treatment had no effect on ROS production in THP-1 cells. **c** 2F5 treatment had no effect on ROS production in Jurkat cells. **d** 2F5 treatment had no effect on ROS production in CEM-C7 cells.

**Fig. S6 ROS inhibitor NAC could reverse the promotion effect of 2F5 on APL cell differentiation.** Differentiation marker CD14 in NB4 and HL-60 cells were analyzed by flow cytometry four days after differential treatment. Cells were divided into six groups: 40μM 2F5 treatment group, 100μM H_2_O_2_ positive control group, 5mM NAC treatment group, 5mM NAC combined 40μM 2F5 treatment group, 40μM IgG treatment group, and untreated normal control group. **a-g** NAC combined 2F5 significantly decreased the CD14-positive NB4 cell percentage compared with NB4 cells treated with 2F5. ***P*<0.01. **h-n** NAC combined 2F5 significantly decreased the CD14-positive HL-60 cell percentage compared with HL-60 cells treated with 2F5. ***P*<0.01.

**Fig. S7 Schematic diagram of hypothesis.** Based on the experimental results obtained in this study, the following assumptions were made. The dotted line represents the speculated hypothesis, and the solid line represents the results confirmed by experiments. **a** 2F5 is speculated to bind to extracellular nucleotide and transports to the cell plasma via ENT2 (arrow ①), and then DDX5 expression level is down-regulated by 2F5 (arrow ②). Subsequently, ROS production is promoted (arrow ③), and APL cells differentiate along the monocytic lineage finally. SiRNA-induced DDX5 depletion exerts similar promotion effect with 2F5 on APL cell differentiation (arrow ④). However, NAC-induced ROS inhibition (arrow ⑤) blocks the inhibition effect of 2F5 on DDX5 (arrow ⑥) and cell differentiation promotion effect of 2F5. **b** Three paths have been demonstrated by experiments: ①2F5 induced APL cell differentiation by inhibition of DDX5 and subsequent production of ROS (Figure 2, 4, 5, 6, 7, 8 S5 and S6); ② Transfection with SiDDX5 mediated similar effect on APL cell differentiation compared with 2F5 (Figure 8); ③ NAC-mediated ROS inhibition also blocks the decreasing effect of 2F5 on DDX5 and subsequent promotion effect of 2F5 on ROS and APL cell differentiation (Figure 7 and Figure S6).
